# Micro computed tomography analysis of barley during the first 24 hours of germination

**DOI:** 10.1186/s13007-024-01266-4

**Published:** 2024-09-16

**Authors:** Olivia Doolan, Mathew G. Lewsey, Marta Peirats-Llobet, Neil Bricklebank, Nicola Aberdein

**Affiliations:** 1 Biomolecular Sciences Research Centre, Sheffield Hallam University, City Campus, Sheffield, S1 1WB UK; 2 La Trobe Institute for Sustainable Agriculture and Food, Department of Plant, Animal and Soil Sciences, La Trobe University, AgriBio Building, Bundoora, VIC 3086 Australia; 3 Australian Research Council Research Hub for Medicinal Agriculture, La Trobe University, AgriBio Building, Bundoora, VIC 3086 Australia; 4 Australian Research Council Centre of Excellence in Plants for Space, La Trobe University, AgriBio Building, Bundoora, VIC 3086 Australia

**Keywords:** Micro-computed tomography, Barley, Grains, Germination, Methodology, Morphology, 3-dimentional, Internal structure

## Abstract

**Background:**

Grains make up a large proportion of both human and animal diets. With threats to food production, such as climate change, growing sustainable and successful crops is essential to food security in the future. Germination is one of the most important stages in a plant’s lifecycle and is key to the success of the resulting plant as the grain undergoes morphological changes and the development of specific organs. Micro-computed tomography is a non-destructive imaging technique based on the differing x-ray attenuations of materials which we have applied for the accurate analysis of grain morphology during the germination phase.

**Results:**

Micro Computed Tomography conditions and parameters were tested to establish an optimal protocol for the 3-dimensional analysis of barley grains. When comparing optimal scanning conditions, it was established that no filter, 0.4 degrees rotation step, 5 average frames, and 2016 × 1344 camera binning is optimal for imaging germinating grains. It was determined that the optimal protocol for scanning during the germination timeline was to scan individual grains at 0 h after imbibition (HAI) and then the same grain again at set time points (1, 3, 6, 24 HAI) to avoid any negative effects from X-ray radiation or disruption to growing conditions.

**Conclusion:**

Here we sought to develop a method for the accurate analysis of grain morphology without the negative effects of possible radiation exposure. Several factors have been considered, such as the scanning conditions, reconstruction, and possible effects of X-ray radiation on the growth rate of the grains. The parameters chosen in this study give effective and reliable results for the 3-dimensional analysis of macro structures within barley grains while causing minimal disruption to grain development.

## Background

With an ever-growing population and the rising risk of climate change, there is an increasing strain on food production. Grains make up a large proportion of both human and animal diets, with barley being amongst the most important [1]. Barley is one of the most cultivated crops worldwide. In 2021, over 145 million tons of barley grain were harvested. Globally, 70% of barley production is used directly or indirectly for animal feed and the remainder is mostly used in malting for the beverages industry [2, 3]. Furthermore, barley is often favored as animal feed due to its high relative protein and nutrient content [4]. Being able to grow successful and sustainable crops is essential to food security in the future.

The barley grains have a complex structure, composed of many tissues and organs. Amongst these, the embryo (diploid) develops into a seedling, and the energy for this transition is provided by the endosperm (triploid). The aleurone layer surrounding the endosperm aids in the uptake of water at the beginning of the germination phase, whilst the scutellum sits between the endosperm and embryo and transports nutrients from the endosperm to the embryo [5, 6]. The embryo itself is composed of several tissues and cell types, each with distinct roles during germination and post-germinative growth, including the plumule (embryonic foliage leaves) and radicles (seminal roots). Germination is considered complete when the radicle first emerges from the seed coat. Considering this we intend to use these important features within the grain to evaluate the rate of germination.

Plants have been bred for grain yield and quality since the foundation of agriculture and the domestication of crops. It is understood that the morphology of grains is important to their success. Historically, grains have been selectively bred based on their external morphological characteristics [7, 8]. However, with the development of technology we can now begin to consider the internal characteristics which may determine successful germination. For example, it is understood that the size and shape of the aleurone layer affects the ability of the grain to uptake water [9]. Moreover, the volume of the endosperm is key to the nutrient reserve of the grain, being able to isolate this organ within the grain will give insight into the grains chance of success and, more specifically to barley, can indicate the malting properties [10]. By expanding our understanding of grain morphology we can apply this knowledge to increase the yield and quality of the resulting crop, improving its sustainability. Formation of the root system is critical for a plant’s growth and development. The roots act as the main intake system for vital nutrients and water, as well as anchoring the plant in the soil. The emergence of the first rootlet from the base of a grain (radicle) is considered the end of the germination phase [11]. This makes them key to understanding the rate of development of germinating barley grains.

Optical light microscopy and histology have been the main methods for visualizing the anatomy of plants [12]. Traditional light microscopy techniques such as fluorescence microscopy, are valuable for investigating cellular and sub-cellular organelles, making it essential for plant research. However, a large amount of sample preparation is often involved. The sample must be sectioned, mounted, and in some cases stained to acquire a 2-dimensional (2D) image of a plant’s lifecycle [13, 14]. This technique can be destructive, as the sample preparation can, in some cases, deform the morphology of the samples being tested. Therefore, being able to visualize, to a high degree of accuracy, the changes in the internal architecture of a plant during its life cycle without destroying the internal microenvironment would expand our cognizance of plant development. Plants are inherently 3-dimensional organisms and reducing them to a small number of sections imaged in 2-dimensions limits our ability to interrogate the fine structure of plant organs and relate this to function. Consequently, visualizing plant organs in 3-dimensions (3D) would broaden our understanding of the architecture and configuration of cells, plant organ development, and how they change over time [15].

In addition to optical microscopy other grain analysis techniques, such as the SC-G automatic seed analyzer, are commercially available. This technique can be used to analyze the surface and weight of grains, giving measurements such as kennel diameter and length [16, 17]. Although, these factors are key to grain development and viability, they give no indication of the internal organ development of grains and can only be indicators of external grain morphology. Therefore, instruments such as the SC-G seed analyzer are very useful tools for high-throughput analysis of overall grain surface area and volume, they cannot record the detail of the morphological changes to organs and tissues within the grain.

Micro Computed Tomography (µ-CT) is an X-ray tomography technique with high resolution that has begun to be applied for 3-dimensional imaging of plant samples [18–20]. It is a non-destructive imaging technique based on the differing X-ray attenuations of a material, performed by using an X-ray tube with cone-beam geometry and a rotating sample holder. The changes in the intensity of the attenuation as the X-ray passes through the sample are described using the Beer-Lambert law [21]. Furthermore, this can be used to establish the relative density of the sample based on the distance from the source and the reduced intensity of the detected X-ray beams. µ-CT has been used and improved extensively since its invention by A. Cormack and G. Hounsfield in 1979. The benefits of this technique have greatly advanced the field of medical imaging and have resulted in the production of guidance on optimizing µ-CT scanning parameters by the medical field. Most importantly, when imaging living tissues with X-rays, the effects of ionizing radiation must be considered, because ionizing radiation has a cumulative effect on living tissue, therefore each additional scan may cause an increase in potential damage to the DNA within the tissue [22, 23]. However, guidelines for appropriate radiation dosages are not available for applications to plant tissues [24, 25].

**Fig. 1:**
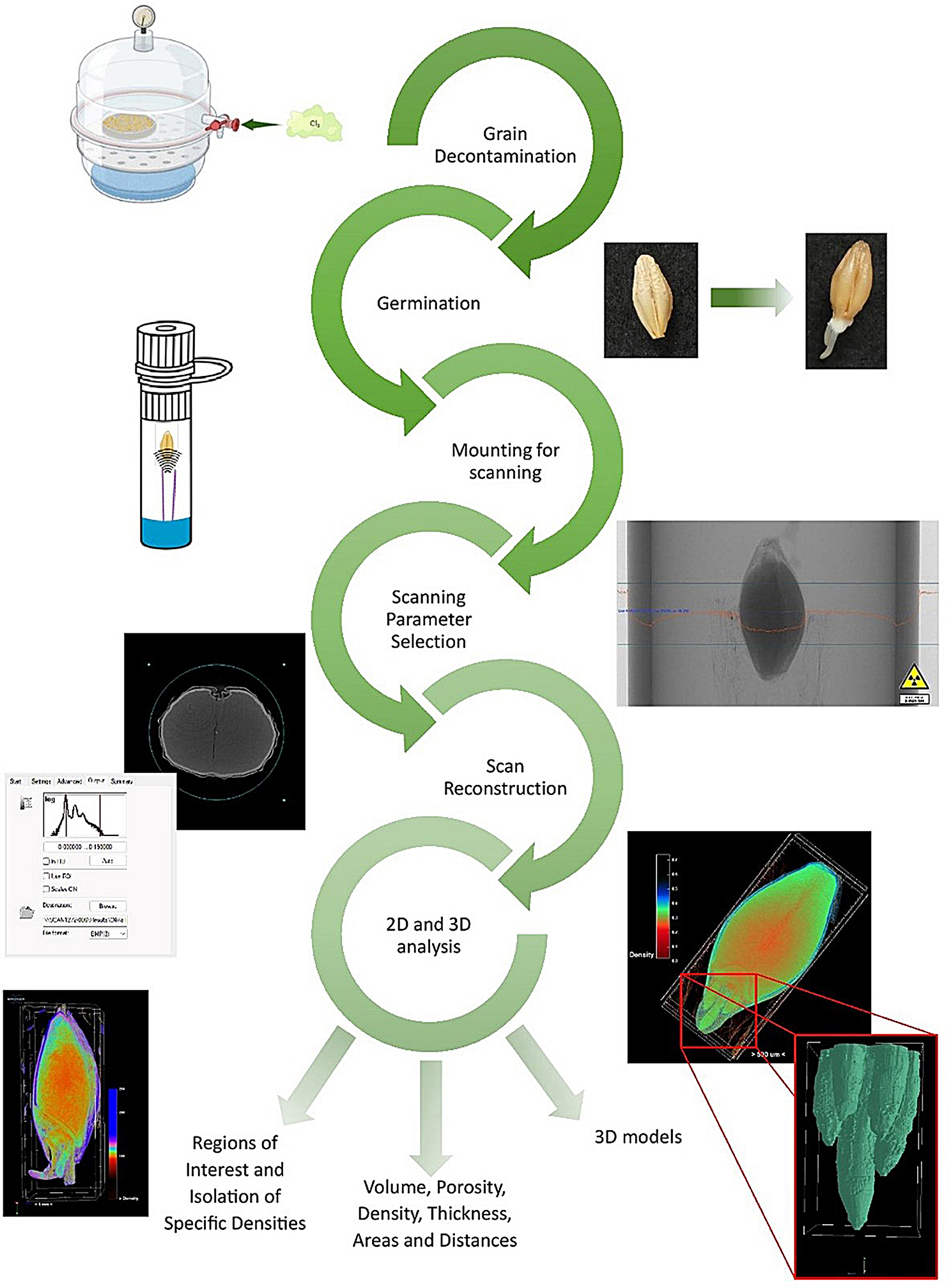
Workflow for imaging of barley grains using Micro-CT, starting with grain sterilization using chlorine gas to remove the risk of infection during germination, the germination and mounting for scanning, the selection of crucial scanning and reconstruction parameters, finally resulting in the possible 2D and 3D outputs which are possible

In our study, we aimed to establish robust and appropriate µ-CT methodology to image barley grains during germination (Fig. 1). The non-destructive nature, high resolution and minimal sample preparation make µ-CT a powerful tool for the in depth analysis of the internal structure of grains. Additionally, µ-CT offers a larger field of view, when compared to traditional microscopic techniques allowing a whole organism to be imaged in detail simultaneously. Previous research has demonstrated the application of µ-CT for studying wheat grains and tomato seeds [26, 27]. L Gargiulo et al. [26]. established a method looking at how many tomato seeds successfully germinated post-scanning, comparing abnormal, dead, and completely un-germinated seeds. The study considered morphological indicators within the seeds that may indicate the likelihood of successful germination [26] A. Suresh et al. [27] used µ-CT to visualize the damage caused by insects to the internal structure of wheat [27]. More recently, techniques such as deep learning have been applied to µ-CT imaging of plant matter allowing the 3D morphology of plant tissue to be accurately resolved on a cellular level [28], further illustrating the power of µ-CT as an imaging technique.

These studies illustrate the potential of µ-CT for imaging of plant material. However, there has been little research that considers the impact of sequential ionizing radiation dosages on the embryo and germination of grains. Our study aimed to optimize µ-CT scanning parameters for the barley grain that enable users to visualize the internal morphology of the grain during the first 24 h of germination with minimal effects on embryo morphology or germination parameters. It is hoped this can be applied to shed light on how specific organs develop within the grains over time.

## Materials and methods

### Plant material and germination

Barley grain (*Hordeum vulgare* cultivar La Trobe) was sourced from the University of Adelaide [29]. Prior to germination, *n* = 66 barley grains were surface sterilized. In brief, the grains were placed in a sealed desiccator unit with a beaker of bleach (3%v/v) to which hydrochloric acid (97%v/v) was added, and the resulting reaction produced chlorine gas (Cl_2_). The grains were left for 2 h in the desiccator, after which the gas was vented and purged with nitrogen gas (N_2_), to displace the Cl_2_. The grains were then placed in a sealed, sterile container at room temperature, ready for germination.

Individual grains were placed in wells of a 6-well plate (ThermoScientific, Nunclon Delta Surface) wrapped in medical tissue (Northwood Hygiene) and 3mL of sterile, deionized water (Millipore) was added at time 0 h. Germination was carried out in a temperature and light-controlled grow chamber (Sanyo, Versatile Environmental Test Chamber). The grains were grown in the dark in the chamber to simulate growth beneath soil and the temperature was set at 23 °C. Analysis was carried out at set time points of 0, 1, 3, 6 and 24 h after imbibition (HAI). The time points were chosen based on studies conducted by Peirats-Llobet et al. [29], and represent a comprehensive spectrum of activity across germination, from early to late.

### Mounting

**Fig. 2:**
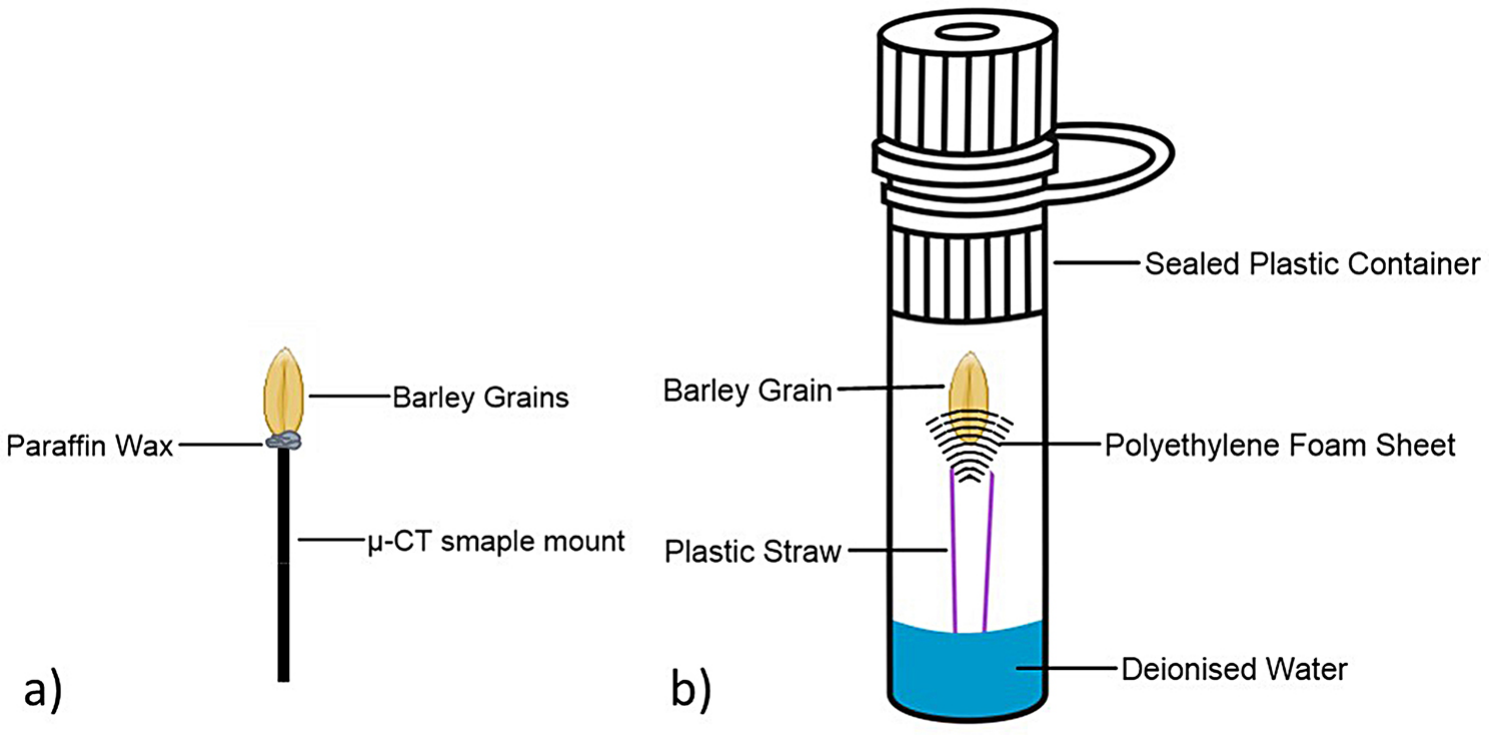
Illustration of scanning set up: (**a**) for dry grain scanning. (**b**) for imaging after imbibition

Each sample (i.e. a single grain) was securely mounted within the sample chamber of the µ-CT before scanning to avoid movement during the scanning process. For dry grains, this required the use of paraffin wax to anchor the grain (Fig. 2a). Wet grains (those in which germination had been initiated) were placed on a pedestal within a sealed plastic container, with a reservoir of water below to avoid dehydration of the sample during the scanning protocol, and with care taken to avoid adhesive attachment that may damage the grain (Fig. 2b).

### Scanning parameters and reconstruction

Scans of the barley grains were acquired using a SkyScan 1272 (Bruker, USA) benchtop µ-CT unit. The SkyScan 1272 has several parameters that can be changed simultaneously to optimize the quantification and quality of the 3D image output. Five scanning parameters and 2 reconstruction parameters were investigated in the development of this method (Table 1). The scanner was controlled using the SkyScan 1272 Control Program (Bruker, Version 1.5.0.0). NRecon (Brucker, Version 2.2.0.6) was used to reconstruct the 3D images acquired from the scanner. Data Viewer (Bruker SkyScan, version 1.6.0.0) was used to orientate the reconstruction and select a specific region of interest (ROI) for analysis. The orientation was centered in the middle of the grain with the sagittal axis running through the crease, the transverse axis across the widest part of the grain, and the coronal axis perpendicular to the sagittal, running lengthwise through the grain.

**Table 1:** List of scanning and reconstruction parameters used during optimization and corresponding citations describing parameter applications in more detail

Scanning Parameters:									Reference
									[24, 30]
Filter	No filter	0.25 mm Al	0.5 mm Al	1 mm Al	0.035 mm Cu & 1 mm Al	0.11 mm Cu			[31–33]
Resolution	5	5.5	9	10	13				[34]
Camera Binning	1008 × 672	1344 × 896	2016 × 1344	4032 × 2688					[35]
Rotation Step (Degrees)	0.2	0.3	0.4	0.5	0.6	0.7	0.8		[34]
Frame averaging	1	2	3	4	5	6	7	8	[35, 36]
**Reconstruction Parameters**:									
Beam hardening	10%	20%	30%	40%					[31, 32]
Ring artifact reduction	1	2	3	4	5	6	7	8	[37]

Cu – Copper, Al - Aluminum

### Camera binning evaluation

Camera binning affects the overall image resolution, with a higher resolution achieved from a greater number of pixels per field of view [35]. To establish the best camera binning and therefore resolution 10 grains were scanned with both the 2016 × 1344 (2 K) camera binning and 4032 × 2688 (4 K) camera binning detectors. The full set of scanning parameters for this experiment has been outlined in Table 2. Scanning parameters are used to optimize camera binning and resolution.

**Table 2:** List of scanning parameters used during the 2 K vs. 4 K comparison scans

Camera Binning	2016 × 1344	4032 × 2688
Resolution	7.5 μm	3.75 μm
Rotation Step	0.3 degrees	0.3 degrees
Frame Averaging	5 frames	5 frames
Filter Selection	No Filter	No Filter
Scan Time	27 min 15 s	49 min 16 s
Data File Size	3.13 GB	12.93 GB

### Post reconstruction analysis

CTAn (Bruker SkyScan, Version 1.21.2.0) was used to process and analyze the reconstructed data set from the selected regions of interest (ROIs). The use of an ROI was employed to analyze the rootlets within the grain embryo.

For the isolation of macro-structures within germinating grains the key operations were: thresholding, despeckling, 3D analysis, and 3D model. 3D models were viewed using the CTVol Software (Bruker SkyScan, Version 2.3.2.3).

### Effects of x-ray radiation

A number of scanning protocols were investigated to determine the effects of x-ray radiation on germination. The first being the ‘Multi scan’ protocol, where a single grain was scanned at each time point (0–24 HAI). The second being the ‘double scan’ protocol, where each grain was scanned at 0 HAI and at each timepoint respectively. Therefore, double scan grains (*n* = 30) were exposed to radiation twice in 24 h whereas multi scan grains (*n* = 6) were exposed to radiation 5 times in the 24 h. A single scan protocol, where each grain was only scanned once, was also proposed but was not used for 3D analysis. The cumulative radiation dose received by the grains was calculated over 5 germination time points (0 HAI, 1 HAI, 3 HAI, 6 HAI, and 24 HAI) for all 3 scanning protocols (*n* = 66).

## Results

**Fig. 3:**
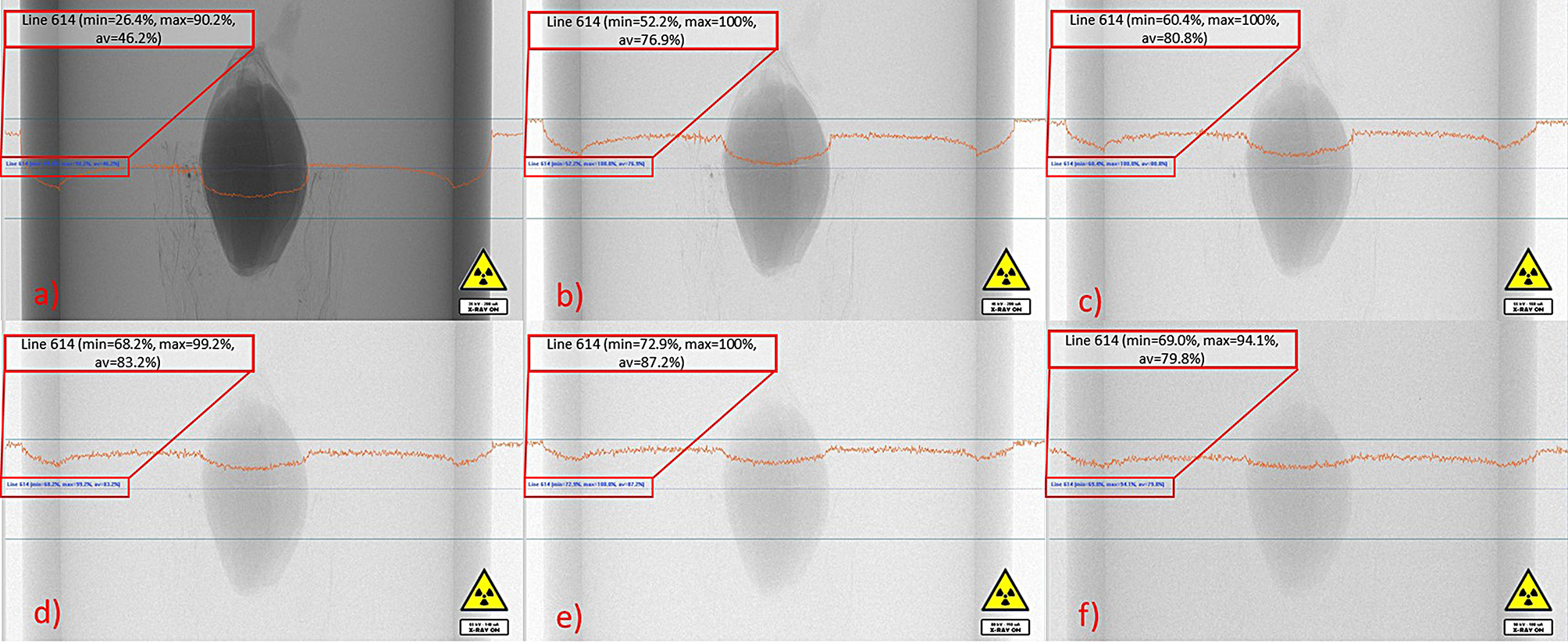
A comparison of possible filters available on the SkyScan 1272, each image shows the flat field, highlighting the maximum, minimum and average attenuation of the x-ray as it passes through the widest part of the sample. (**a**) no filter, (**b**) 0.25 mm aluminum, (**c**) 0.5 mm aluminum, (**d**) 1 mm aluminum, (**e**) 0.035 mm copper and 1 mm aluminum, (**f**) 0.11 mm copper

### Scanning parameters

#### Filter selection

We first determined the appropriate filter for our analyses. We tested 6 filter options by assessing the attenuation of the grain at the 0 HAI timepoint [31, 32]. Figure 3 shows the levels of attenuation of the X-ray as it passes through the grain. It is well accepted in the field that the minimum attenuation of the beam should be no less than ~ 30% and the maximum no more than ~ 95%, when passing through the middle or most dense area of the sample. With no filter the minimum attenuation is 26.4%, the maximum is 90.2% and the average is 46.2%. Alternatively, when the next available filter is applied (Aluminum 0.25 mm) the minimum is 52.2%, the maximum is 100% and the average is 79.9%. The contrast in the image continues to decrease as the filter absorption capacity increases. Using the final filter, copper 0.11 mm, the lack of contrast can be seen in Figure 3, with a minimum attenuation of 69.0%, and a maximum attenuation of 94.1%. Therefore, the optimum beam attenuation through the grain was achieved using no filter, this gave the closest maximum and minimum values to that generally accepted within the field.

#### Rotation step and frame averaging

We tested several frame averages and degrees of rotation to establish the most appropriate combination for our study. As illustrated in Table 3, there is a difference of 53 min from the lowest to highest combination of rotation steps and frame averages available. If we decrease the degree of rotation by 4 fold from 0.8 to 0.2, keeping the frame averaging at 1, we increase the scan time by 3 fold, from 7 to 23 min. Whereas, if the average frames are increased by 8 fold, from 1 to 8 frames per step, the scan time increases from 23 to 60 min (3 fold). Additionally, decreasing the rotation from 0.8 to 0.2 results in an increase in file size approximately 4 fold from 1.18 GB to 4.70 GB, increasing the storage and processing power required for the scan files. Considering these factors, we settled on 0.4 degrees of rotation and 5 average frames per step which resulted in a 23-minute scan and 2.35 GB data generation.

**Table 3:** List of rotation step (degrees) and average frame options, comparing the data load and scan times for each combination of parameters

	*Rotation Step (Degrees)*							
		*0.2*	*0.3*	*0.4*	*0.5*	*0.6*	*0.7*	*0.8*
Frame averaging (number of shots per rotation step)	1	23 min / 4.70GB	17 min / 3.13GB	13 min / 2.35GB	9 min / 1.88GB	9 min / 1.57GB	7 min / 1.34GB	7 min / 1.18GB
	***2***	*30 min / 4.70GB*	*20 min / 3.13GB*	*15 min / 2.35GB*	*11 min / 1.88GB*	*10 min / 1.57GB*	*8 min / 1.34GB*	*7 min / 1.18GB*
	***3***	*35 min / 4.70GB*	*23 min / 3.13GB*	*18 min / 2.35GB*	*13 min / 1.88GB*	*11 min / 1.57GB*	*10 min / 1.34GB*	*9 min / 1.18GB*
	***4***	*40 min / 4.70GB*	*27 min / 3.13GB*	*20 min / 2.35GB*	*15 min / 1.88GB*	*13 min / 1.57GB*	*11 min / 1.34GB*	*10 min / 1.18GB*
	***5***	*45 min / 4.70GB*	*30 min / 3.13GB*	*23 min / 2.35GB*	*17 min / 1.88GB*	*15 min / 1.57GB*	*13 min / 1.34GB*	*11 min / 1.18GB*
	***6***	*50 min / 4.70GB*	*33 min / 3.13GB*	*25 min / 2.35GB*	*19 min / 1.88GB*	*16 min / 1.57GB*	*14 min / 1.34GB*	*12 min / 1.18GB*
	***7***	*55 min / 4.70GB*	*37 min / 3.13GB*	*28 min / 2.35GB*	*21 min / 1.88GB*	*18 min / 1.57GB*	*15 min / 1.34GB*	*14 min / 1.18GB*
	***8***	*60 min / 4.70GB*	*40 min / 3.13GB*	*30 min / 2.35GB*	*24 min / 1.88GB*	*20 min / 1.57GB*	*17 min / 1.34GB*	*15 min / 1.18GB*

#### Resolution and camera binning

We determined the camera binning and resolution combination which was most appropriate for the scanning of grains is 2 K. Factors that were considered were the scanning time, image quality, the data outputs, and further processing of the data. Increasing the image resolution, without changing the field of view, can be achieved by increasing the camera binning. By increasing the camera binning of the scan from 2 K to 4 K, the scan time and file size approximately double. With a scan time of 27 min at 2 K camera binning versus 49 min at 4 K camera binning. Additionally, the file size produced is 4 times larger for the 4 K scan compared to the 2 K scan.

**Fig. 4:**
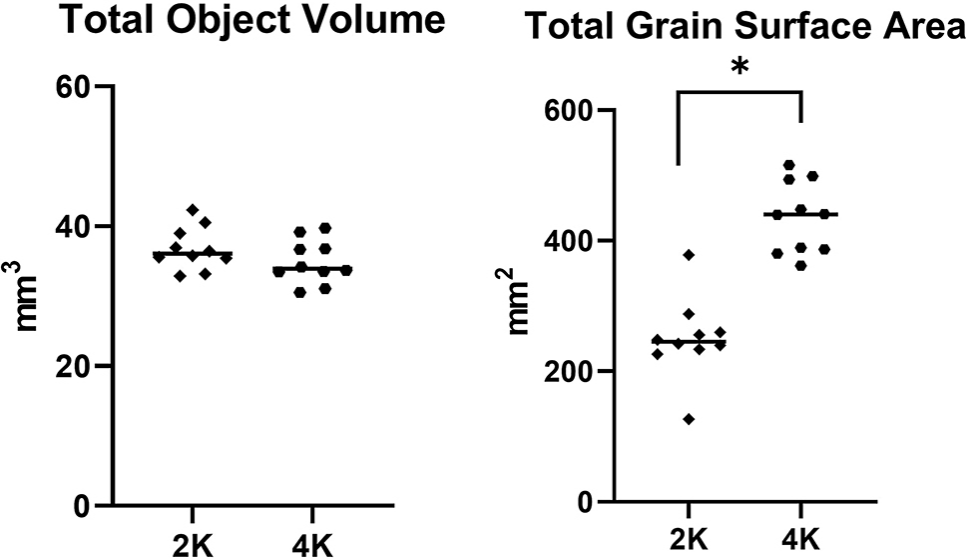
Comparison of 2K and 4K camera binning, the post 3D analysis data. Total object volume showing no significant difference between the data sets (p= 0.18). The total grain surface area shows a significant difference between the data sets (*P = <* 0.001)

Post-3D analysis, the volume and surface area of the grains were compared (Fig. 4). Although there is little variation in the volume of the grains between the 2K and 4 K scans (*P =* 0.18), there is a significant difference between the surface areas with the surface area of the 4 K grains being nearly 200 mm^2^ more than those scanned with the 2K camera binning (*P = <* 0.001*).*

**Fig. 5:**
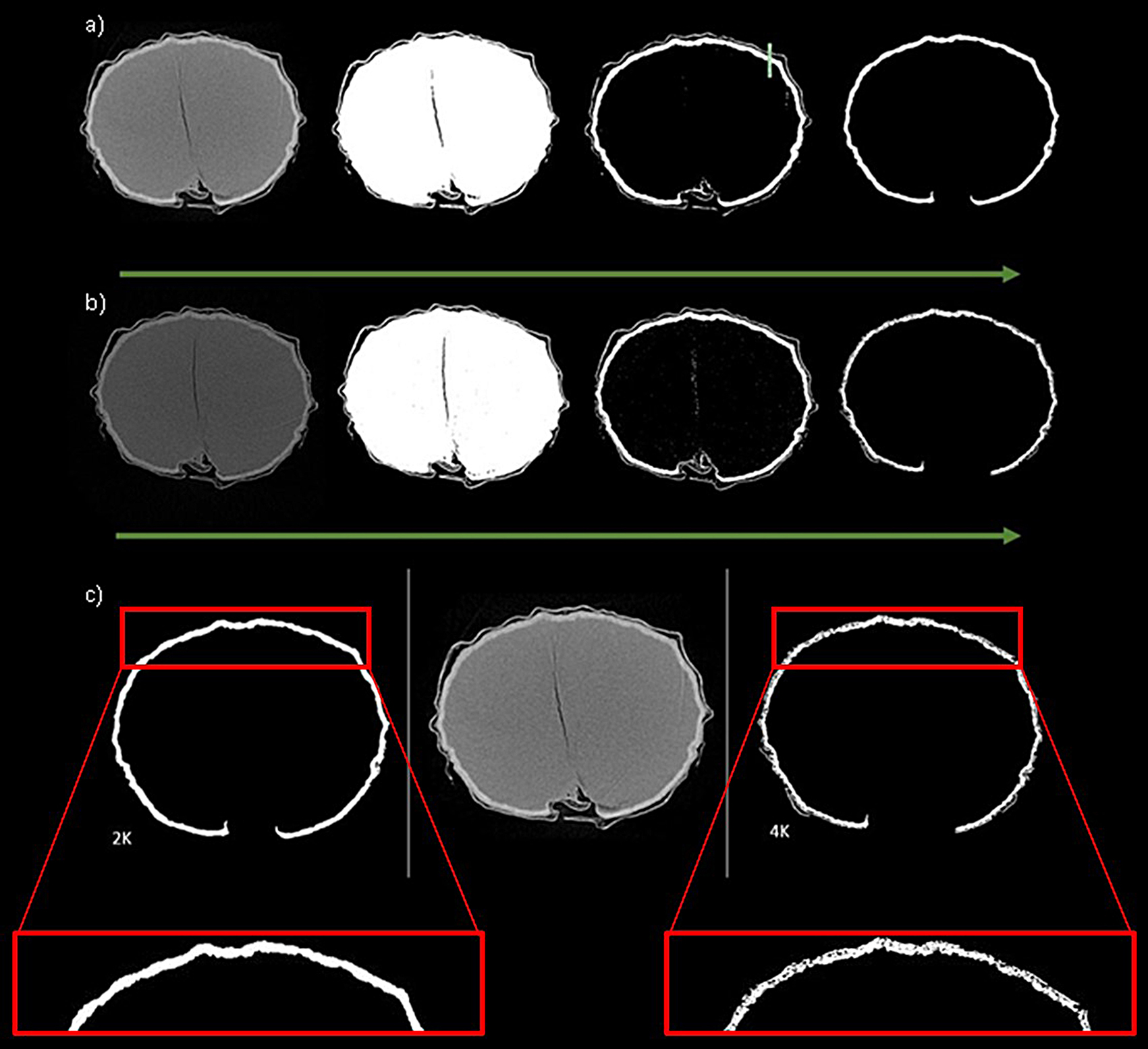
Thresholding in CTAn of grains scanned using 2K and 4K camera binning to identify most appropriate time vs. image quality and resolution. (**a**) using the 2K camera binning, images left to right, starting with x-ray image of a grain cross section, binarized image isolating the whole grain, binarized image isolating the aleurone layer by density, post despeckling function, removing husk/leaving the largest object which is the aleurone layer. (**b**) using 4 K camera binning, left to right, starting with x-ray image of a grain cross section, binarized image isolating the whole grain, binarized image isolating the aleurone layer by density, post despeckling function, removing husk/leaving the largest object which is the aleurone layer. (**c**) side by side comparison of the isolated aleurone layer comparing the result when using 2K camera binning (left), 4K camera binning (right) and an Xray image (center) for comparison

This can be visualized with the binarized images acquired during thresholding. As an example of binarized isolation, the aleurone layer is used. The binary images of the grains imaged with 4K camera binning show pitting and holes throughout the image when trying to isolate specific regions of interest. Figures 5 a) and b) show the steps taken to binarize and isolate the aleurone layer for a), 2K camera binning and b), 4K camera binning scans. Figure 5 c) shows a side-by-side of the x-ray and binarized images. Acquired at 4K camera binning results in excessive pores within the image which may not necessarily be significant when analyzing macro structures within the sample. Therefore, this high level of detail is not required for the analysis of macro structures within the grains and can prove to be a hindrance when post-acquisition analysis takes place.

### Reconstruction parameters

**Fig. 6:**
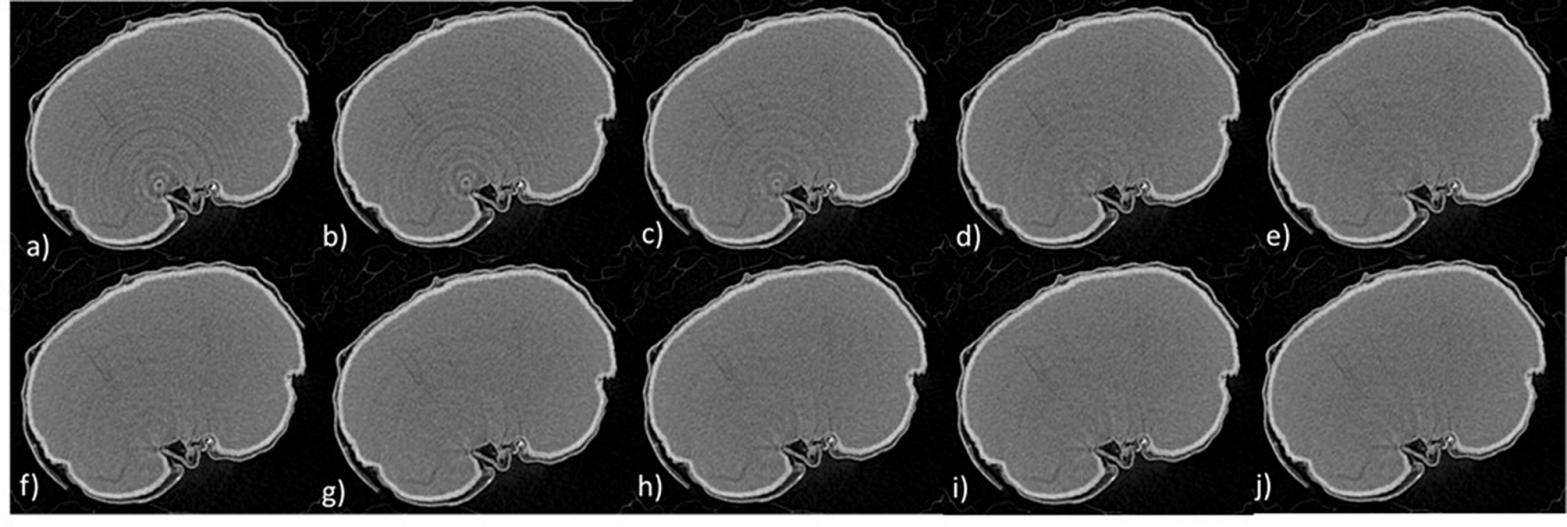
Ring artifact reduction - a central slice of a barley grain with increasing levels of ring artifact correction (**a**-**j**) starting with no correction applied (**a**) and increasing up to (**j**). After (**h**) there is no apparent improvement in the quality of the image as further correction is applied. Making this the most applicable correction parameter

#### Ring artifact reduction

Correcting artifacts of scanning can be achieved with the ‘ring-artifact reduction’ feature within NRecon. It is well established within the field that a ring artifact correction of between 1 and 10 is acceptable. Figure 6 illustrates the reduction of ring artifacts as this parameter is increased. Until image h) the ring artefact is reduced as the correction is increased. However, in i) and j) the correction factor is increased but no further improvement can be seen in the image. A ring artifact reduction setting of approximately 8 was selected for these samples. However, this can be adjusted per scan to allow for the most accurate representation of the sample, avoiding under or over-correcting.

**Fig. 7:**
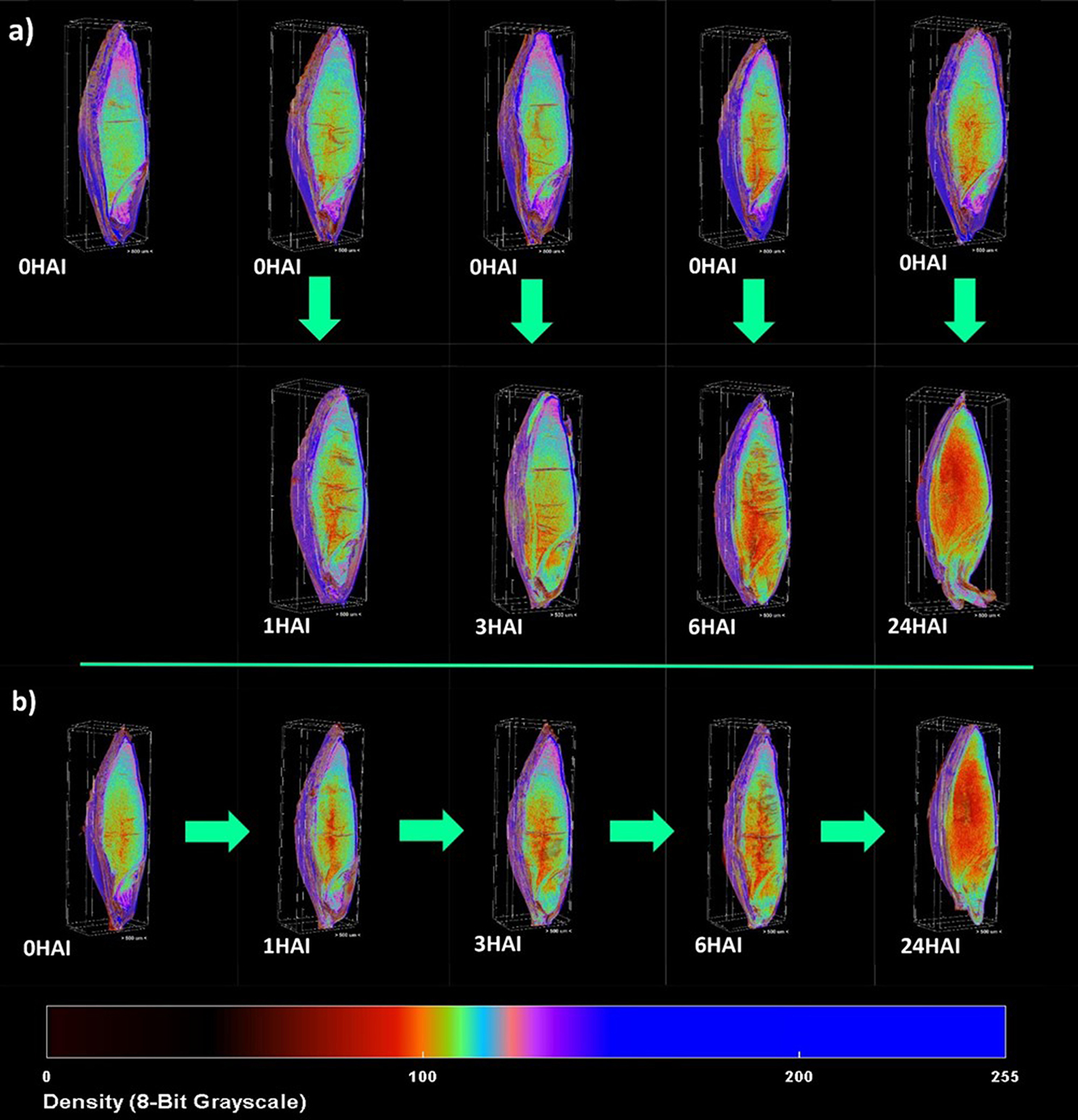
µ-CT images of (**a**) double scan grains which were scanned at 0 HAI, then at set timepoints and (**b**) a singular grain which was repeatedly scanned at each set timepoint. The pseudo coloring represents comparative density

### Effects of x-ray radiation

**Fig. 8:**
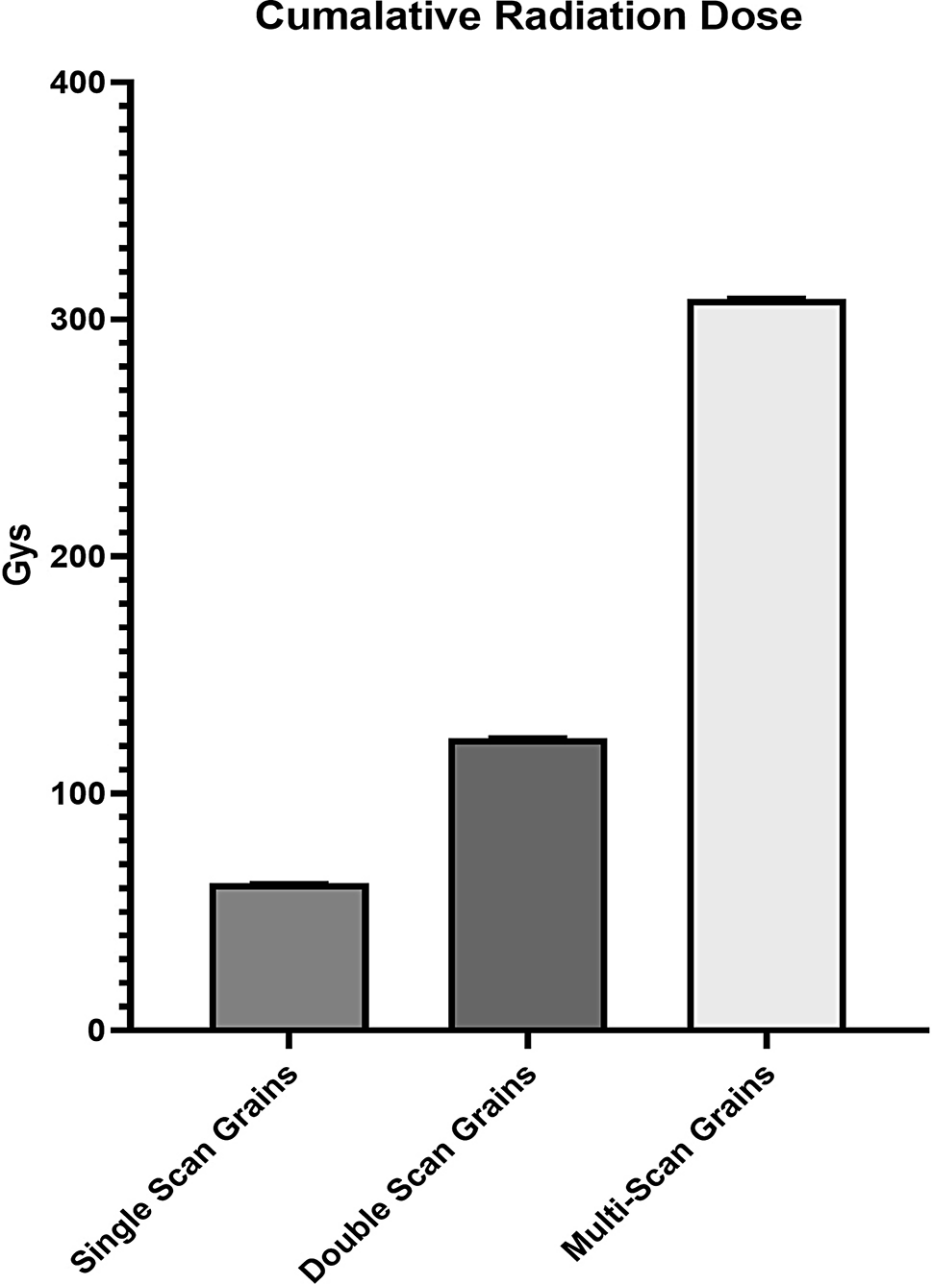
Graph showing the differences in cumulative radiation received by grains scanned just once, grains scanned twice and grains scanned at every timepoint. The graph illustrates the 5 fold increase in radiation dose received by the multi scan grains when compared to the single scan grains

Figure 7 illustrates the changes that occur across the time points, visualizing the different scanning protocols used in this study. The dose of ionizing radiation given to each grain must first be calculated before its effects can be established (Fig. 8). The dose received by the double scan grains is twice that received by the single scan grains, with the double scan grains receiving 123.6 Gry on average and the single scan grains receiving only 62 Grys during the scan. Furthermore, the multi-scan grains receive a dose of 308.5 Grys, 5 fold more than that of the single-scan grains.

**Fig. 9:**
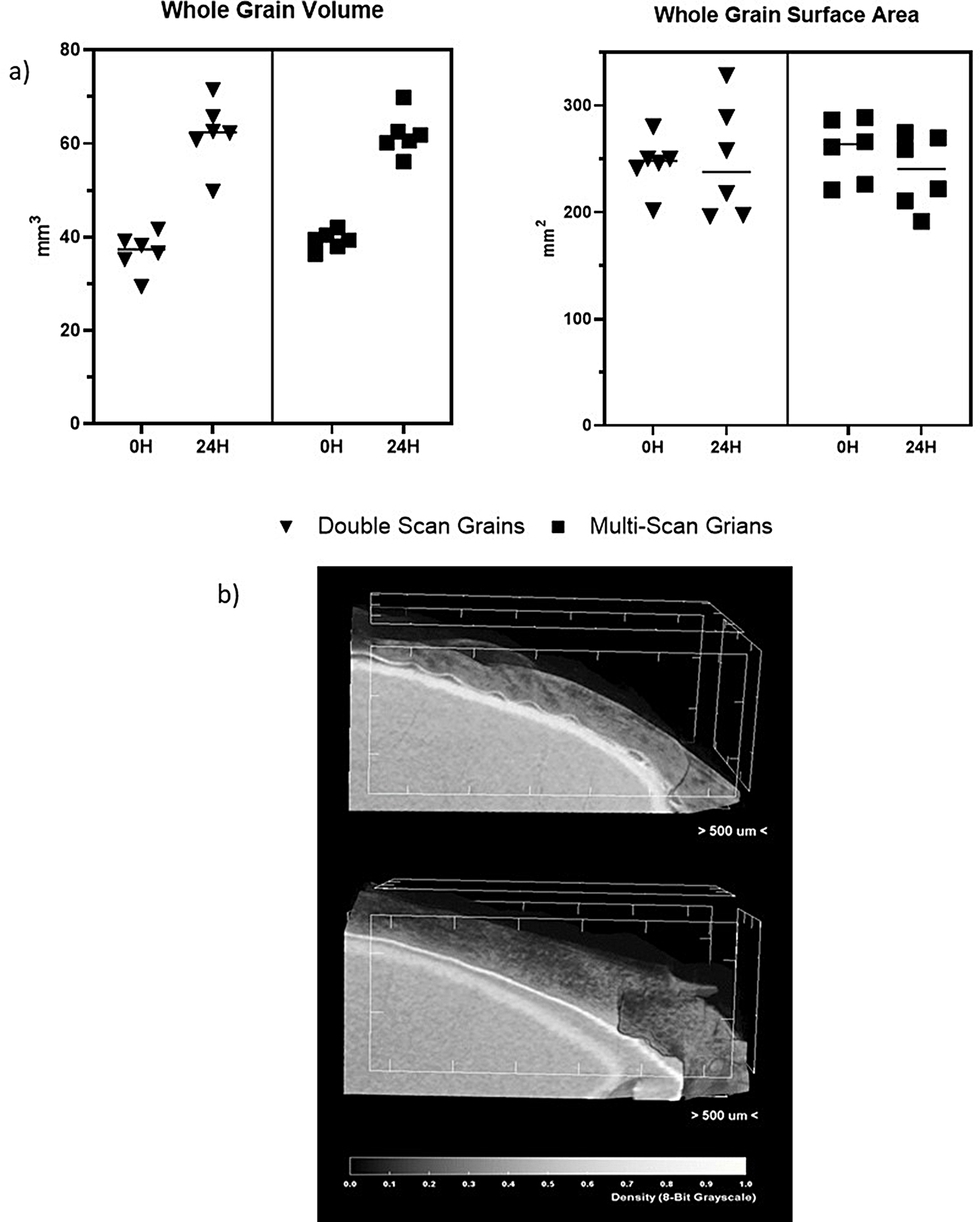
(**a**) Graphs showing the change in total volume and total surface area over the first 24 h of germination. The graph illustrates the difference between the double scan and multi scan grains, showing that there is no significant difference between the 2 scanning conditions. (**b**) Close up cross section of a grain at 0 HAI (top) and 24 HAI (bottom), showing the changes in the surface of the grain over time

When evaluating the possible effects of radiation, one key measurement for comparison was the overall grain volume increase after 24 h (Fig. 9a). The double-scan grains and multi-scan grains appeared to have no difference in the overall grain volume despite the difference in radiation dose. With the double-scan grains having an average increase of 25.39 mm^3^ and the multi-scan grains having an average volume increase of 22.60 mm^3^ (*P* = 0.259). Suggesting that the uptake of water and grain swelling after imbibition is unaffected by the µ-CT radiation in the first 24 h. Interestingly, there is no significant change in the surface area of the grains between 0 and 24 h. With the average change in surface area for double-scan grains being 2.76 mm^2^ and the change in multi-scan grains being − 20.35 mm^2^ (*P* = 0.385). The surface area of each grain is ~ 250 mm^2^, on average each grain appears to only undertake a small fluctuation in overall surface area in the first 24 h of germination. This appears to be due to the nature of the husk of the dry grain, and how it expands as water is taken up. This can be visualized using CTVox where the husk becomes visibly less wrinkled over time (Fig. 9b).

**Fig. 10:**
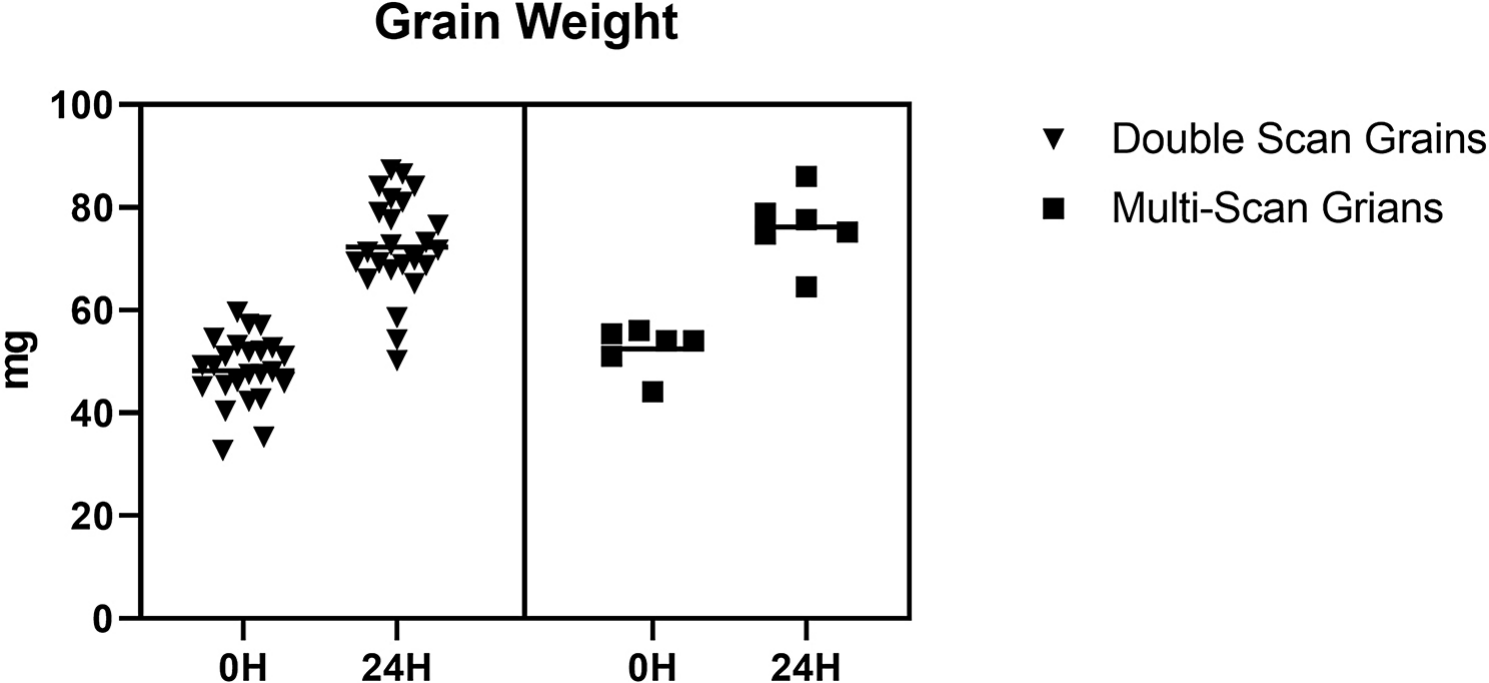
Graph showing the increase in grain weight 24 HAI, the graph shows no significant difference between the double scan and multi scan grain weight change. (*p* = 0.868)

The weight of the grain was monitored as a possible indicator of radiation effects (Fig. 10). The weight increased by an average of approximately 25 mg during the experiment and there was no significant difference between the double scan and multi-scan grains (*P=* 0.868). The overall size and shape changes of the grains showed that there is little difference between scanning the same grain multiple times or limiting the scanning to just twice.

**Fig. 11:**
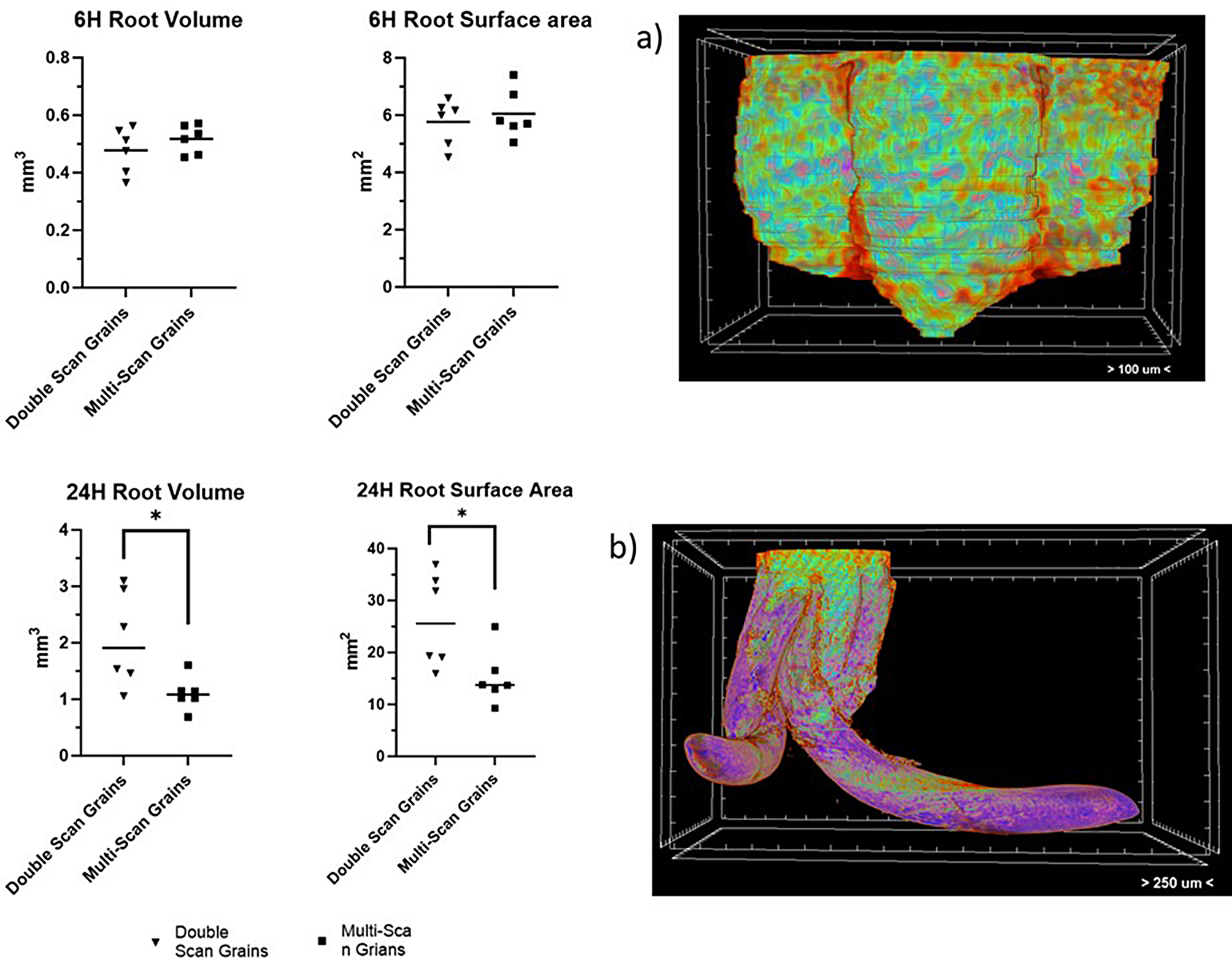
(**a**) 6 HAI root volume and surface area, comparing multi scan and double scan grains, showing no significant difference between the scanning protocols. Image a is a CTVox image of isolated root system at 6 HAI. (**b**) 24 HAI root volume and surface area, comparing multi scan and double scan grains, showing a significant difference between the scanning protocols. Image b is a CTVox image of isolated root system at 24 HAI

The volume and surface area of the rootlets were analyzed after 6 and 24 h to establish if growth was being affected and when, in the timeline, this became apparent. Figure 11a shows the volume of the pre-emerged rootlets at 6 HAI and Fig. 11b shows the radical at 24 h. These volumes have been isolated using the CTAn software. At the 6 HAI, there was no significant difference between the surface area (*P =* 0.558) or volume (*P* = 0.327) of the multi-scan rootlets compared to the double-scan rootlets. The multi-scan rootlets had an average surface area of 6.06 mm^2^ and a volume of 0.52 mm^3^. The double-scan rootlets had an average surface area of 5.76 mm^2^ and a volume of 0.48 mm^3^. However, at 24 HAI, we saw a significant difference between the rootlets that were scanned twice to those that were scanned multiple times. At 24 HAI, the average surface area of the multi-scan rootlets was 15.20 mm^2^ and the average area of the double-scan rootlets was 26.16 mm^2^ (*P* = 0.028). The average volume of the rootlets was 2.07 mm^3^ for the double-scan rootlets versus a volume of 1.11 mm^3^ for the multi-scan rootlets (*P* = 0.025).

## Discussion

The aim of this study was to optimize µ-CT scanning parameters of barley grains to visualize the internal morphology during the first 24 h of germination. The selection of the optimum µ-CT scanning parameters is linked to the quality of the image obtained. Many variables are interlinked and must be optimized to improve the quality of the final image. Altering specific settings at the scanning stage will affect the parameters required for reconstruction and image analysis. This study has revealed that the optimum scanning parameters for a barley grain can be based on 2 K camera binning, no filter, a rotation step of 0.4 degrees, and a frame averaging of 5 frames.

### Filter selection and beam hardening

Photon attenuation and scattering are two of the most critical factors in achieving high-quality imaging whilst minimizing scan parameter artifacts. Attenuation and scattering result in the surface of the sample to appear more dense than it truly is [24]. The main causes of photon scatting are the Compton effect and the Photoelectric effect, common phenomena when imaging soft tissue [38]. To reduce the effects, filters have been designed to enrich the beam by absorbing lower energy photons resulting in a ‘hardening effect’ on the beam. However, in applying a filter to a scan the intensity of the beam is reduced, which in turn decreases the signal-to-noise ratio. This decrease in intensity can result in a loss of contrast within the sample. Although these parameters have been explored by other groups using different grains and a variety of filters [39–41], our study shows no filter to be optimal for imaging barley grains, selecting this based on values closest to the minimum (30%) and maximum (95%) attenuation. This is specific to both the power of the x-ray withing the µ-CT and the density of the grain in question, making this a variable that will change between grain species and the instrument used.

The filter selected for scanning directly influences the level of beam hardening correction required. Filters are a known way to ‘harden’ the X-ray beam withing a µ-CT. This is achieved by the filter absorbing lower energy photons before they pass into the chamber. These lower energy photons are responsible for the photoelectric and Compton scattering which make the exterior of the sample appear more dense [31, 30]. For the reasons outlined above, we selected no filter, this meant the ‘hardening’ benefits of the filter were lost. The manufacturer recommends a setting between 10 and 40%. When not using a filter at the scanning stage there is an increased likelihood of the scattering phenomenon occurring, resulting in the correction being on the higher end of the manufacturer’s suggestion [34].

### Rotation step and frame averaging

When choosing the rotation step and frame averaging, we considered the acquisition time, image quality and data load. The SkyScan 1272 uses a step-and-shoot scanning method, whereby the sample turns a set number of degrees and an image is captured before turning again. The images are then knitted together during the reconstruction process. When selecting the rotation step, multiple factors must be considered, the first being the image quality. The smaller the degree of rotation in theory the better quality an image will be as each turn focuses on a smaller section of the grain [34]. However, the time required to scan and the size of the data set produced must be considered. A scan with a rotation step of 0.2° will take twice the time to acquire than that with a rotation step of 0.4°. Additionally, twice the amount of data will be acquired making the processing power and data load much greater. The step-and-shoot method introduces the possibility of a small amount of movement in the sample as it turns and stops, this can introduce blurring and artifacts within the scan. One way this can be overcome is by using frame averaging. This will increase the number of images acquired at each step and average them to create a more accurate representation of the sample and minimize the effects of sample movement when scanning. However, this can also have an impact on the acquisition time of the images. L. Gargiulo et al. [26]. and S. Liao et al. [39] use a rotation step of 0.2°. L. Gargiulo illustrates the morphological indicators in pregerminated (dry) tomato seeds, they report a scan time of 54 min, which is suitable for their protocol of scanning [26]. However, this scan time was too long for the germination timeline outlined in the current study. S. Liao et al. [39] outlined a protocol for the analysis of maize kennels. Although they have not outlined the scan times, they do state that 900 projection images were acquired per scan which is almost double the 481 projection images produced with our protocol, suggesting nearly double the amount of processing power required for further image analysis. As the germination time points were an hour apart at their shortest, it was not ideal to have the grains out of the controlled growing chamber for an extended period. Therefore, the rotation step and frame averaging were optimized for 0.4° and 5 frames. When scanning dry grains, the scan times could be extended as the negative effects of germination were not a consideration.

### Resolution and camera binning

Binning refers to the combination of pixel matrices to create one larger pixel, doing this leads to greater contrast and less noise [40]. However, there are limitations as altering the binning of the camera, the resolutions are also affected. The resolution achieved using the 4 K camera is greater than using the 2 K camera binning. By doubling the binning capacity of the camera from 2 K to 4 K, the resolution doubles but the magnification of the sample remains the same. This allows the samples to be analyzed in the same field of view [40, 42]. After considering the results of the 2 K vs. 4 K scans, it was decided that the added detail acquired at 4 K did not outweigh the negative effects of additional artefacts observed when thresholding. Additionally, when considering the nature of the germination study, the extended scan times at 4 K led to negative impacts on the growth rate of the grains. A scan that may take several hours runs the risk of either killing the grain/stunting the germination process or being of poor quality as the grain actively grows, or possibly dies within the chamber, causing smudges and shadowing within the image. S. Dhamgaye et al. [43] observed the effects of ionizing radiation on the common bean, they concluded that the effects are dependent on the stage of growth of the seedling. They looked at water uptake, acid phosphate activity, and shoot and root length, concluding that overall growth rates and enzyme functions were impaired in radiated seeds. S. Dierickx et al. [44] investigate the relationship between resolution and imaging requirements. They have outlined 4 different resolutions for scanning, studying 17 wood species for potential identification. The higher resolution images provided greater detail, however, there are limitations associated with the field of view available for scanning and detail required for the scan purpose. Therefore, outlining the considerations made when selecting an appropriate resolution for scanning and how the nature of the sample and output required can affect the resolution required.

### Reconstruction

Raw 2D projection images are obtained from the scanning process, showing the attenuation of the x-rays within the sample. The individual 2D rotation image projections must be stacked to create the 3D reconstruction. The software used to achieve this was NRecon. All images reconstructed using NRecon are visualized over 255 shades of grey, with black being the least dense material within the sample chamber (air), to white being the most dense material within the chamber. The software uses the data collected and plots a logarithmic histogram of the signal acquired. The histogram plots relative intensity against the X-ray attenuation coefficient. Using the feature within the software, it is possible to tell the reconstruction over what attenuation signals to assign the 16-bit greyscale or the 255 shades of grey. To make the density of separate scans comparable the x-ray attenuation co-efficient must remain identical from scan to scan. Multiple factors must be considered when reconstructing a sample, such as post-alignment, smoothing, beam hardening, and ring artifacts, using features in the software the parameters can be altered to allow for the reconstruction to be as true a reflection of the sample as possible.

Beam hardening corrections is a tool within the NRecon software that corrects for Compton scattering and the photoelectron effects caused by low-energy photons [31, 32]. This phenomenon can make the surface of a sample appear more dense than reality. It is well known withing the field that a beam hardening correction between 10 and 50% is acceptable depending on the scanning conditions and sample. For this study, it was determined that a beam hardening correction of around 30–40% was used, through a visual assessment of the reconstruction.

Much of the current work using µ-CT to image grains does not discuss the parameters of beam hardening and ring artifact correction this is likely due to the variability in corrections needed from instrument to instrument and scan to scan [27, 37, 45]. The use of these settings is often dictated by visual inspection of images and determining the levels of correction required. However, it is essential to use these corrections effectively as they can influence the output of data processing. For example, a method for the quantitative analysis of wheat grains has been described previously, but the beam hardness parameters are not given, and ring artifacts are present in the images generated [40, 45]. It should be noted that if this is not corrected properly it can lead to distortion of the images when further analysis takes place.

One reconstruction parameter to be aware of is the post-alignment compensation. This function measures how far from the center of rotation the sample has swung when rotated during the scan. For optimal µ-CT scanning the sample should be a perfectly cylindrical tube that is exactly perpendicular to the X-ray beam. A sample of this nature would require no post-alignment, this is difficult to achieve in reality so to ensure the scan slices line up correctly, post-alignment must be applied. This is predicted and applied automatically by the software, NRecon, but can be manually adjusted and is assessed on a scan-by-scan basis.

Another factor that must be considered when reconstruction is the smoothing. Smoothing is for noise reduction within the image. However, excessive smoothing can affect the level of detail you can see within the scans. Using the smoothing feature in NRecon you can select the number of pixels you wish to smooth. For this study, we selected 1 pixel, this removed any excessive noise without interfering with details in the image. This was selected by referring to previous guidelines and literature [24, 46], although centered around bones and biomaterials, the reasoning behind applying smoothing in this way applies to our study.

### Image analysis and thresholding

The use of regions of interest (ROIs) is employed to isolate areas within grains. Due to the nature of the tissue within grains it is not always possible to isolate areas based on density alone, an ROI must be established to segment the scan before further analysis can take place. This technique was applied to analyze the embryo within the grain. Although the density of the embryo is similar to the rest of the grain it can nonetheless be seen clearly as a separate organ within the internal structure. Therefore, to isolate this structure an ROI was carefully drawn around the edge of the image of the organ before further morphological functions could be applied such as thresholding, despeckling, erosion and accretion, 3D analysis, and rendering of 3D models.

Thresholding is a method of segmentation where images are segmented into two different classes of pixels, foreground, and background. The image is then binarized into the desired image and the background based on the threshold value set with the grey-level histogram of the image. This can be done on a slice-by-slice basis or globally across the whole scan [47]. When optimizing the binning, thresholding plays a key role in deciding the most appropriate settings. Then considering the desired analysis outcomes, which for this study is isolating macrostructures within the grains, having a high camera binning and therefore a high resolution can be a hindrance to the analysis process. Excessive detail from high-resolution scanning can cause issues when isolating densities of interest.

3D models were viewed and analyzed using CTVox. As far as analyzing growth rates and patterns in developing grains is concerned, this may not initially seem to be the most relevant output from this data. However, advances in technology and biomaterials have increased the demand for large databases of X-ray CT Scans [15]. Currently, there are few open-access databases dedicated to plant structures and organs. X-Pant is a relatively new publicly available database containing X-ray datasets and models. Databases such as these, aim to corroborate several useful outputs that can be valuable to the scientific community and contribute to further research [48].

### Effects of radiation

The impact of radiation on the germination rate was initially established visually. The grains that had been in the µ-CT during initial optimizations scans were stunted, the radicle was emerging comparatively later than the grains which had not been exposed to the radiation. However, a protocol to establish this hypothesis needed to be tested.

When considering radiation dose in living tissues, there has been extensive research carried out in the medical field [22, 25]. From this research, there are specific weightings dependent on the tissue types that indicate the absorbed doses of radiation. This is considered the effective dose and is an indicator of potential damage caused by the ionizing radiation [23]. However, this has not been established for all living tissue, such as the absorbed dose in plant tissue. Due to this knowledge gap, we can only estimate the potential dose given to the barley grain during the scanning process. To be able to accurately calculate the dose for a sample as small a grain would be challenging. In mouse samples, a dosimeter is placed within a PMMA tube of 25 mm thickness to estimate the depth-corrected dose and then placed within the mouse within the organ of interest. However, with the width of a grain typically being only 3 mm across the feasibility of this kind of measurement is not practical [25]. Therefore, for this study, we used the maximum possible absorbed dose, which is assumed to be equivalent to the dose received by the air within the chamber at the distance from the source where the grain is positioned. From this, we can calculate the maximum cumulative dose received by grains based on scan times and the emissions from the X-ray source. The radiation dose received by the air within the chamber was calculated using the Bruker software CTion. CTion is based on the simulation of x-ray photon energy spectra and dose by the executable SpekCalc. The software calculates radiation dose in milli-greys per minute (mGy/min) in air based on the current and voltage of the source, the distance to the source and the type of filtration applied between the sample and source [42, 49]. These doses offer a comparative figure, allowing us to semi-quantify the different possible doses received by each grain. The calculation of radiation dose in living tissue is complex, with many contributing factors. Factors that require specialized equipment and technique to determine, such as interference from scanning the mounting conditions, the specific tissue weighting of the grain tissue, and how this might change as the gains uptake water and develop. From these calculations, it is clear that the grains that were scanned multiple times received a significantly higher dose of radiation than those scanned once or twice.

Development of the root system is critical for a plant’s growth and development. The roots act as the main intake system for vital nutrients and water, as well as anchoring the plant in the soil. The emergence of the first rootlet from the base of a grain (radicle) is considered the end of the germination phase [11]. This makes them key to understanding the rate of development of germinating barley grains. µ-CT facilitates in-depth 3D analysis of the root system before it has begun to emerge. This root development can also be used as an indicator of the effects of radiation.

Overall, there is an indication of some damage caused by the radiation after multiple scans. It is also important to consider other contributing factors such as the ambient temperature of the laboratory and the amount of time spent outside of the growing chamber. It is difficult to determine whether the effects on growth are directly caused by the radiation dose, localized heating from the radiation, or the disruption to the grain environment caused by removing it from the growth chamber for imaging. However, we cannot ignore the accumulation of radiation dose caused by multiple scans and how this affects the outcome of the germination process. Using different grains for each scan eliminates the limiting factor of radiation dose and possible disruption to growing conditions caused by removing the grains multiple times from the grow chamber, allowing for more flexibility in scanning parameters. We have focused on macro structures withing the grains, however, if scanning requires greater detail and therefore longer scan times, greater negative effects may be seen. Considering these factors, we propose that scanning grains dry (0 HAI) and at representative time points (1 HAI, 3 HAI, 6 HAI, 24 HAI) is the ideal protocol for the study of germinating barley grains.

## Conclusion

Imaging live plant tissues is still a relatively unexplored area of µ-CT. In this work we have optimized and established the ideal scanning conditions for the 3D analysis of grain size, volume, and surface area on germinating barley grains. Furthermore, we have confirmed the utility of µ-CT technology to examine macro structures within the developing grains. We also identified a number of limiting factors that have been considered including the drying of grains during scanning and the possible effects of radiation on the rate of germination. We present some key scanning conditions such as including the mounting for scanning being enclosed to avoid drying, the required resolution vs. scanning time and data workload, and how the camera binning can affect the later processing of the data. For barley, we have determined, to minimize any possible negative effects by imaging the grains just twice, once at 0 HAI and at respective time points, resulting in less disruption to the growing environment and limiting possible effects from radiation. Here, we lay the foundations of a reliable method for the effective imaging of germinating barley using 3D µ-CT, a non-destructive method that has the potential to expand the study of plant development.

## Data Availability

The datasets used and/or analysed during the present study are stored in the Sheffield Hallam SHURA data repository and can be made available by contacting the corresponding author upon request.
